# Integrative evidence-informed management of feverish illnesses in childhood—outcomes of the public FeverFriend prospective app registry

**DOI:** 10.3389/fpubh.2026.1757745

**Published:** 2026-04-09

**Authors:** Henrik Szőke, Jan Vagedes, Anikó Balogh

**Affiliations:** 1 Department Integrative Medicine, Faculty of Health Sciences, University of Pécs, Pécs, Hungary; 2 Department of Neonatology, University-Hospital, Tuebingen University, Tuebingen, Germany; 3 ARCIM Institute (Academic Research in Complementary and Integrative Medicine), Filderstadt, Germany; 4 School of Social Sciences and Mannheim Business School, University of Mannheim, Mannheim, Germany

**Keywords:** antibiotic stewardship, antipyretic use, behavioral change, evidence-informed fever management, integrative pediatrics, mobile public health program

## Abstract

**Background:**

In conventional mainstream practice there is a prevailing negative attitude towards fever, which persists despite the evidence of beneficial immunological effects and lack of practical implementation of this knowledge into the healthcare system. Integrative approaches represent an added value in promoting behavioral change towards supportive fever management. We examined associations between use of the FeverFriend^®^ app and caregiver-reported antibiotic and antipyretic use and healthcare utilization for febrile illnesses in children.

**Methods:**

Prospective patient registry within a mobile app, including a decision aid, connected with a knowledge base. Recruitment and data collection were conducted continuously throughout the study period from 01 December 2020 to 1 September 2025. In this publication we evaluate Hungarian users’ data. The study population ranged from otherwise healthy individuals to patients with chronic diseases, aged between 0 and 18 years. There were no pre-specified groups or subgroups assigned to receive specific intervention(s) (or no intervention) based on the study protocol.

**Results:**

In total, 24,371 caregivers registered 26,042 children. Caregivers recorded 17,994 illness episodes and 31,874 temperature measurements; 7,377 episodes included a completed end-of-illness review. Paired analyses were based on children with both baseline and reviewed-episode data. Caregiver-reported antibiotic use, antipyretic use, and doctor consultation were lower in the reviewed episode than in baseline recall (McNemar test with continuity correction, all *p* < 0.001).

**Conclusion:**

FeverFriend registry data are consistent with an association between app use and lower episode-level reporting of antibiotics, antipyretics, and doctor visits compared with 12-month baseline recall.

## 1 Introduction

Fever is a common symptom among children, presenting a challenge in its management for caregivers (1). Numerous physiological, pathophysiological, and clinical evidence support the beneficial effects of fever in augmenting immune defense (2, 3, 4, 5). In fact, elevated body temperature may provide an advantage in controlling microbial infections compared to aggressive antibiotic and antipyretic interventions (6, 7, 8, 9).

However, in mainstream practice there is a prevailing negative attitude towards fever (10), which persists despite the clinical and scientific evidence (11, 12, 13). Sociological research suggests that this suppressive attitude is primarily rooted in two factors: the desire for comfort and fear of fever, despite the numerous benefits associated with allowing fever (14, 15).

In contrast, traditional, complementary and integrative approach’s attitude towards fever management is more likely to be permissive or supportive, including lower antipyretic and antibiotic use (16, 17, 18).

Based on this facts, a research agenda was set up and published to highlight the added value of Traditional, Complementary, and Integrative Healthcare (TCIH) to the WHO and the WHO/FAO/UNEP/WOAH 2023 Research Agendas on Antimicrobial Resistance, including the FeverFriend app as a favorable research topic (19).

Research gap shows up in limited real-world evidence on how digital decision-support tools influence caregiver behavior and healthcare utilization in everyday pediatric fever management. In particular, large-scale, prospective data capturing real-time caregiver decisions outside clinical settings remain scarce. This study addresses this gap by examining behavioral associations related to the use of a pediatric fever management app within a national app-based registry.

Current professional guidelines show significant variations of recommendations (20). There is a lack of practical implementation of up-to-date and integrative knowledge among both healthcare professionals and the lay public although two latest guidelines include integrative-supportive fever management recommendations (21, 22).

Therefore, the FeverFriend project aims to promote evidence-informed guideline adherence by reducing unnecessary use of antipyretics and antibiotics, as well as alleviating the burden on the healthcare system by reducing frequency of doctor contacts. Based on the latest NICE guidelines (23) clinicians have developed a protocol and register called FeverFriend program. This program (containing the FeverFriend mobile phone application among other elements) allows parents and caregivers to document fever symptoms and runoff, while receiving feedback on patients’ state risk assessment including a complex algorithm—developed in a Delphi process of specialists with 100% consensus—resulting in an RAG protocol (high-risk = red, intermediate = amber, low-risk = green) and a decision aid for appropriate management (see Appendix B for caregiver advice).

An independent international “Systematic Assessment of Pediatric Fever Management Apps” rates the FeverFriend app as one of the three best evidence-based fever management apps with parental decision support systems worldwide (24).

The program and its protocol was involved in the Emergency Service’s Platform and the development of the new guideline on fever management in children in Hungary (21).

The general aim of this study was to examine associations between the use of a digital pediatric fever management application and caregiver behavior in real-world settings. Specifically, the study aimed to assess changes in management of childhood febrile illnesses, using data from a prospective app-based registry. By analyzing self-reported, real-time data, the study seeks to contribute evidence on the potential role of digital decision-support tools in promoting evidence-informed fever management and reducing overmedicalization.

The objective of the presented study is to investigate the association between use of the FeverFriend app and antibiotic and antipyretic use, as well as the frequency of utilization of healthcare services by caregivers of children.

## 2 Materials and methods

### 2.1 Study design

We conducted a prospective, adaptive, cohort, observational patient registry within a mobile app, utilizing Ecological Momentary Assessment.

The prospective nature of the study design allowed us to collect data over time, capturing information on participants as they progressed through the study period. This approach enabled the assessment of changes and outcomes that occurred during the course of the study.

The adaptive feature of the study design allowed for flexibility in data collection and analysis. It allowed us to modify data collection protocols, measurements, and assessments based on emerging findings, ensuring that the study remained responsive to the evolving research questions and situations.

Furthermore, the longitudinal design enabled the examination of a specific group of individuals who shared common characteristics or experiences. By following this group over time, we were able to observe and analyze various factors related to fever management and healthcare utilization.

As an observational study, our focus was on observing and documenting participants’ behaviors and outcomes without intervening or manipulating any variables. This approach allowed us to capture real-world data in a naturalistic setting, providing valuable insights into the experiences and practices of caregivers regarding fever management.

Ecological Momentary Assessment (EMA) is a widely used research method in the field of health behavior to gather real-time data on individuals’ measurements, behaviors, and experiences in their natural environment (25, 26). It involves collecting data from participants through repeated sampling of their experiences in real-time or near real-time, typically using electronic devices such as smartphones.

The primary goal of EMA is to capture momentary fluctuations in individuals’ health states, behaviors, and contextual factors, providing a more accurate and ecologically valid representation of their experiences compared to traditional retrospective self-report measures. By collecting data in real-time, researchers can minimize recall bias and capture events and experiences that may otherwise be missed or distorted when relying on retrospective recall alone.

Typically, participants are prompted to respond to brief questionnaires or assessments at specific time points or in response to specific events or triggers throughout their daily lives. EMA allows researchers to examine within-person variability, identify patterns, and contextual factors that influence individuals’ experiences, providing valuable insights into healthcare consumption and behavior.

### 2.2 Study population/recruitment/data collection

Recruitment and data collection were conducted continuously throughout the study period from 01 December 2020 to 1 September 2025. In this publication we evaluate the data of Hungarian users. Participants were included if they or their caregivers downloaded and registered the mobile application, explicitly accepting the legal notice, which included the study protocol, privacy policy, and data management procedures.

Registration required completion of a user profile and creation of at least one patient (child) profile.

Because the app is publicly available and participation is voluntary, the study is based on a non-probability, self-selected (volunteer/convenience) sample of app users. Inclusion criteria for the registry were: (i) caregiver registration with consent to data use; (ii) creation of a patient profile; and (iii) patient age between 0 and 18 years. Exclusion criteria for the registry were minimal by design; however, children with severe chronic disease conditions were excluded from the analytic dataset to increase clinical comparability for fever episodes. Severe chronic disease was identified from the caregiver-entered a field describing chronic conditions in the child’s profile. Free-text entries were coded prior to analysis; records were excluded when the description indicated a serious long-term condition likely to substantially alter infection risk or fever course.

In addition, records were excluded from analyses if they lacked essential information for the respective outcome (see below), were duplicates, or were internally inconsistent.

### 2.3 Sample—description and evaluation of population coverage

The study included a total of 24,371 self-registered users (caregivers) of the FeverFriend app. The users (caregivers) entered 26,042 patients (children) under the age of 18. A total of 31,874 feverish events (measurements) were recorded within the app for patients under the age of 18. 17,994 illnesses were reported, out of which 7,377 were reviewed in detail with completed illness period, where the user provided case specific feedback on the course of the illness. An illness period ends after 48 h, if no further measurement is recorded. All measurements were self-reported by the caregivers.

As for the sociodemographic measures the users provided, the average number of children was 1.64, slightly higher than the Hungarian total fertility rate of 1.39 in 2024 (source: Hungarian Central Statistical Office, https://www.ksh.hu/stadat_files/nep/hu/nep0006.html, accessed on 22 November 2025).

Regarding anthropometric measures, the average age was 3 years and 1.01 months, with a median age of 23.95 months. This represents the typical age distribution of community-acquired infections.

The share of female patients is 48.67%, which is in line with the percentage of females in the Hungarian population aged 3 (source: https://www.ksh.hu/interaktiv/korfak/orszag.html, accessed on 22 November 2025). Additionally, the average weight is 15.23 kg. Comparing this to the World Health Organization (WHO) Child Growth Standards, which indicate an average weight of 14.2 kg for children aged 3 years and 1 month (source: WHO, https://www.who.int/tools/child-growth-standards/standards/weight-for-age, accessed on 22 November 2025), it shows a slight tendency towards overweight. This finding aligns with the results of the WHO European Childhood Obesity Surveillance Initiative for Hungary in 2009–2010, which reported a 25–28% prevalence of overweight children aged 6–9 years in Hungary (source: https://gateway.euro.who.int/en/indicators/cah_64-overweight-and-obese-6-9-years-old/, accessed on 22 November 2025).

The average height in our sample is 96.14 cm, indicating consistency in the measured heights compared to the WHO-reported value of 97 cm for the relevant age group (source: https://www.who.int/tools/child-growth-standards/standards/length-height-for-age, accessed on 22 November 2025).

Additionally, 1.94% of the patients indicated the presence of a chronic disease as significant cardiovascular or respiratory disease, hypertension, Brugada syndrome, chronic renal failure, cerebrovascular disease, hematopoietic/immunodeficiency disease, malignancy, chemotherapy/immunosuppressive/systemic steroid, major overweight, diabetes mellitus, or ectodermal dysplasia.

### 2.4 Analysis populations and data flow

We conceptualized the FeverFriend registry as having four observational units: caregiver accounts, child profiles, feverish illness episodes, and reviewed illness episodes (episodes with a completed end-of-illness review). The headline registry counts therefore differ from the analytic samples used for inferential testing because post outcomes are available only for reviewed episodes.

**Figure 1:**
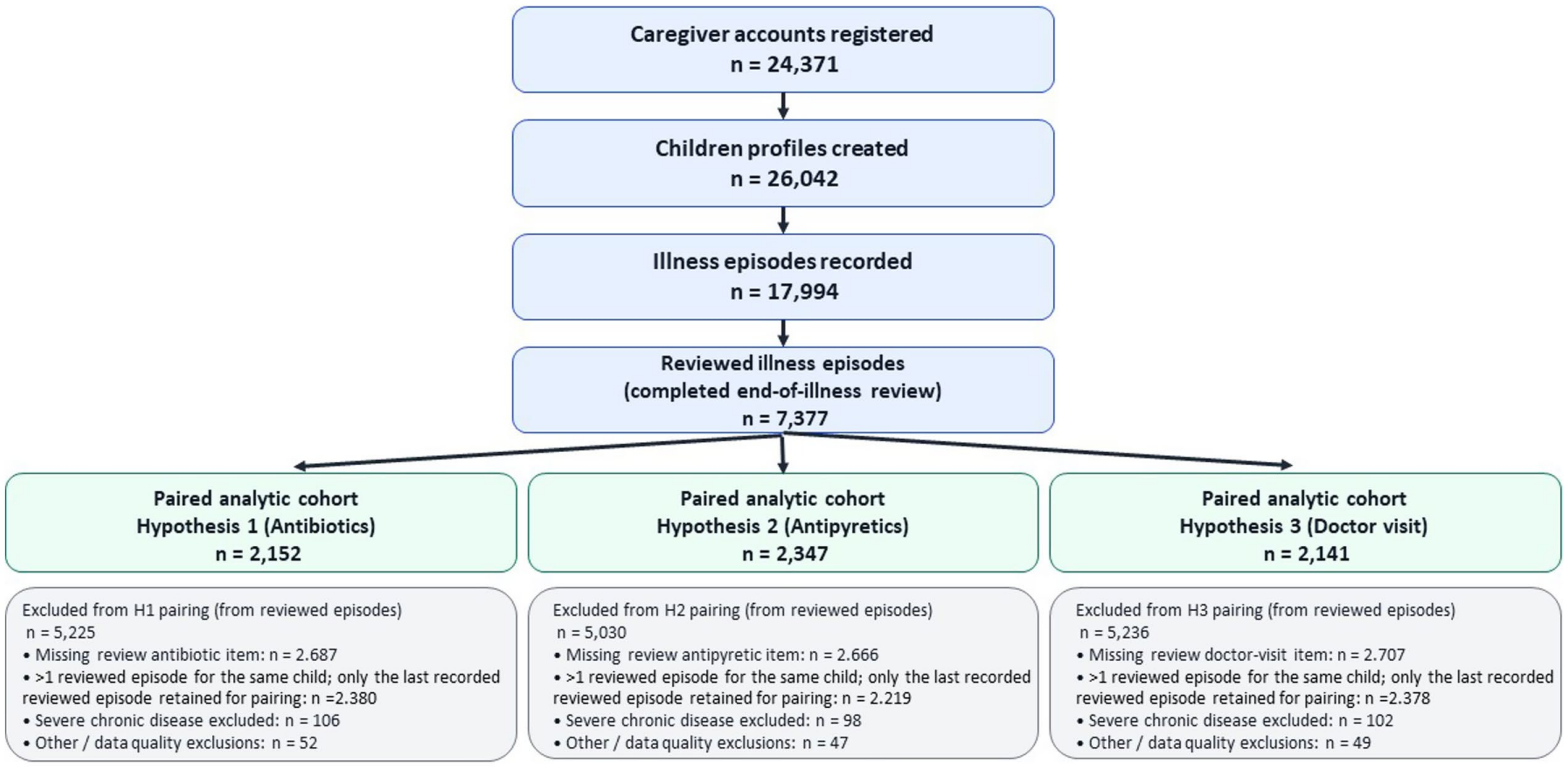
STROBE-style flow diagram of the FeverFriend registry records from registration to final analytic cohorts for Hypotheses 1–3.

For Hypotheses 1–3, inferential analyses were restricted to children with (i) baseline responses on the respective endpoint (12-month recall at child-profile creation) and (ii) the corresponding endpoint reported for a reviewed illness episode. To ensure that each child contributed only one observation per endpoint, if a child had multiple reviewed episodes, we retained only the last recorded reviewed episode and excluded earlier reviewed episodes from pairing. Children were excluded if they met prespecified clinical exclusions (severe chronic disease) or if endpoint-specific post data were missing or invalid. Figure 1 links each step to the exact endpoint-specific paired Ns reported in the Results.

As a descriptive gap analysis, we compared child-level characteristics of children with any complete paired baseline–reviewed data used in the paired analyses with those of children who contributed baseline data only but lacked any completed paired follow-up. This comparison was based on baseline registry variables available in both groups, namely age, sex, and chronic disease status, and was intended as a general assessment of potential selection into the paired analytic subset.

### 2.5 Intervention

The FeverFriend program consists of (1) a mobile application that includes features for registration, documentation of patient’s profiles, documentation of fever events, following personalized advice for both patients and the caregivers, and documentation of follow up notifications. The FeverFriend app is a publicly available, non-commercial mobile health application. (2) An internet-based knowledge base integrated within the app. Both components are available in multiple languages, free of charge and ensure anonymization of user data.

The FeverFriend app provides caregivers with personalized homecare fever management recommendations based on the risk assessment of the patient’s illness. Additionally, the app includes reminders for status administration and encourages caregivers to regularly monitor the child’s status, including body temperature and antibiotics or antipyretics administration.

Advice to caregivers regarding different patient conditions, based on the risk assessment determined by the RAG protocol, can be found in Appendix B. The appendix provides guidance on appropriate management strategies for caregivers to follow based on the assessed risk level of the patient.

### 2.6 Measures

For this study, to observe the change in drug use, we compared the patient’s baseline data on drug consumption in the last 12 months from the time the patient was added to the user profile. Specifically, we analyzed the following variables: “Did you give your patient antibiotics in the last 12 months during a feverish illness?” (variable: ‘usedAntibiotics’) and “Did you give your patient antipyretic drugs in the last 12 months because of fever?” (variable: ‘usedDrug’).

We compared this baseline data to the outcomes of the actual feverish illness in terms of drug consumption, using variables such as “Did [patientName] receive antibiotics during this illness?” (variable: ‘antibioticsMedicationReview’) and “Did [patientName] receive antipyretic medication during this illness?” (variable: ‘antipyreticMedicationReview’).

In the context of antibiotic usage, the application initially presents a binary query regarding the administration of antibiotics. Subsequently, an open-ended question prompts caregivers to input the precise name of the administered medication. During the evaluation of these provided medication names, caregivers often demonstrated a tendency to classify a wider spectrum of pharmaceuticals as antibiotics. Consequently, measures were taken to clean for this measurement error. Entries were standardized by converting to lowercase, trimming whitespace, removing punctuation and diacritics, and harmonizing common spelling variants. Classification was then performed against a national medication list used as the reference dictionary. Medication names were coded using prespecified rules to identify systemic antibacterial agents. Combination products were classified as antibiotics if they contained a systemic antibacterial component. Ambiguous entries were handled using predefined decision rules (e.g., “penicillin” was coded as an antibiotic; non-specific formulations such as “spray” were not coded as antibiotics unless an active ingredient indicating a systemic antibacterial was provided). Coding was completed by a single coder, with adjudication by a clinician for uncertain cases. As a quality check, a random sample of coded entries was independently reviewed by a pediatrician/clinician. Entries that could not be classified with sufficient certainty were coded as missing (2.1% of the entries) and cleaning and recoding procedures resulted in corrections for 9.4% of the entries.

Similarly, we analyzed variables such as “Did you visit a doctor with your patient in the last 12 months because of fever?” (variable: ‘gotToDoctor’) and “Did you consult with a doctor because of [patientName]'s illness?” (variable: ‘gotToDoctorReview’) to compare the utilization of medical provider services.

Baseline items (at child-profile creation) asked caregivers whether the respective behavior occurred at least once during any feverish illness in the preceding 12 months. In contrast, follow-up outcomes refer to the behavior during a single reviewed illness episode recorded in the app. These windows are not directly comparable; thus paired differences should be interpreted as associations between 12-month recall and subsequent episode-specific reporting rather than as episode-matched change.

Standardized app engagement metrics were not available in analyzable form for the present study. Although the registry contains certain process-related fields (e.g., counts of entered measurements, numbers of recorded episodes, and timing of review completion), these were not treated as valid engagement measures because they may also reflect illness severity, episode duration, recurrence, reminder-related behavior, or completion structure rather than engagement with the intervention itself. Core engagement dimensions such as time spent in-app, knowledge-base access, and content-level interaction were not available in a standardized form and were therefore not incorporated.

A detailed list of primary and other outcome measures are provided in Appendix A.

### 2.7 Statistical analytical method

To compare the three baseline variables mentioned above (see 2.5 Measures) with the outcome of the last feverish illness, paired pre–post differences in dichotomous outcomes were assessed using McNemar’s test with continuity correction (two-sided), using the asymptotic *χ*^2^ approximation with 1 degree of freedom. For transparency and reproducibility, we report the discordant pair counts from each paired 2 × 2 table (*b* = baseline ‘yes’/follow-up ‘no’; *c* = baseline ‘no’/follow-up ‘yes’) alongside the test statistic and *p*-value. This test is commonly employed in medical research to compare the frequency of an outcome before and after treatment (27).

In the context of McNemar’s test, a pair refers to two related observations, such as two measurements taken on the same individual before and after treatment. Each pair comprises a “before” value and an “after” value.

To address multiple testing and to control the risk of false positives, we applied the Bonferroni correction. We set the desired significance level (alpha) to 0.05. The adjusted significance level for Bonferroni correction was calculated as alpha divided by the number of tests conducted. After correction, the individual *p*-values were compared to the adjusted significance threshold.

## 3 Results

Our objective was to examine whether significant differences exist in the distribution of certain descriptive categorical variables before and after the intervention, i.e., using the FeverFriend app. Specifically, we investigated changes in three dimensions of healthcare consumption:

1. the use of antibiotics during feverish illnesses; 2. the use of antipyretics for fever management; 3. whether the caregiver seeks professional medical help (emergency unit, ambulance service, doctor) due to fever.

Because Hypotheses 1–3 require paired baseline and reviewed-episode responses for the same child, the analytic sample size differs by outcome; the STROBE flow diagram (Figure 1) provides the full accounting.

To examine possible selection bias into the paired analytic subset, we conducted a descriptive child-level comparison between children with any completed paired baseline–reviewed data and children who contributed baseline data only but had no completed follow-up pair. The groups were compared on age, sex, and chronic disease status (Table 1). The two groups were substantially similar in these observed characteristics, suggesting limited observable selection into the paired analytic subsample.

**Table 1:** Descriptive gap analysis comparing children with any complete paired data versus children with baseline data only.

Variable	Children with any complete paired data (*N* = 2,368)	Children with baseline data only (*N* = 26,042)
Age, mean (SD)	3 years 11.82 months (3.26)	3 years 1.01 months (3.30)
Female sex, N (%)	1,159 (48.34)	12,674 (48.67)
Chronic disease present, N (%)	48 (1.96)	505 (1.94)

*Hypothesis 1*: Changes in the use of antibiotics

**Figure 2:**
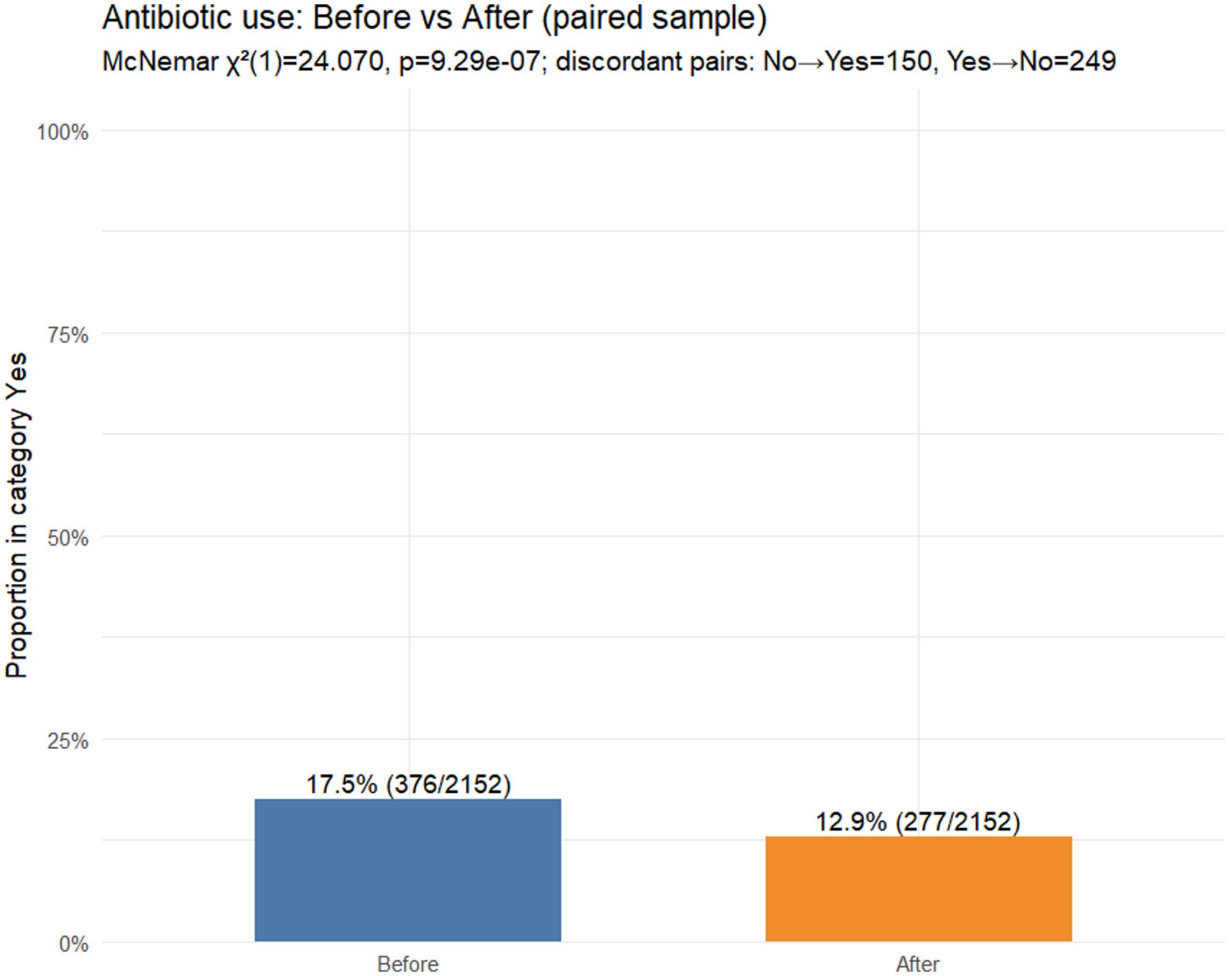
Antibiotics use before vs. after FeverFriend use (paired sample, *N* = 2,152).

In the paired sample (*N* = 2,152), antibiotic use was reported at baseline (12-month recall) by 17.5% (376/2,152) compared with 12.9% (277/2,152) during the reviewed illness episode (Figure 2). Discordant pairs were *b* = 249 (yes **→** no) and *c* = 150 (no → yes), corresponding to a paired risk difference of −4.6 percentage points (95% CI − 6.4 to −2.8) indicating a net shift towards lower antibiotic use. The paired change was statistically significant (McNemar’s *χ*^2^(1) = 24.07, *p* = 9.29 × 10^−7^).

Bars show the proportion of caregivers reporting antibiotic use (“Yes”) at baseline (last 12 months) and in the reviewed illness episode. *p*-value from McNemar’s test based on paired observations.

Using the caregivers’ variables to input the specific antibiotic names yielded an identical outcome: prior to utilizing the app and following the intervention, instances of doctor consultations due to feverish illnesses (occurring either in a pediatric surgery, hospital, or via phone) culminate in a 23% likelihood of antibiotic prescriptions, which is in line with previous data from other western European countries (28).

*Hypothesis 2*: Change in use of antipyretics

**Figure 3:**
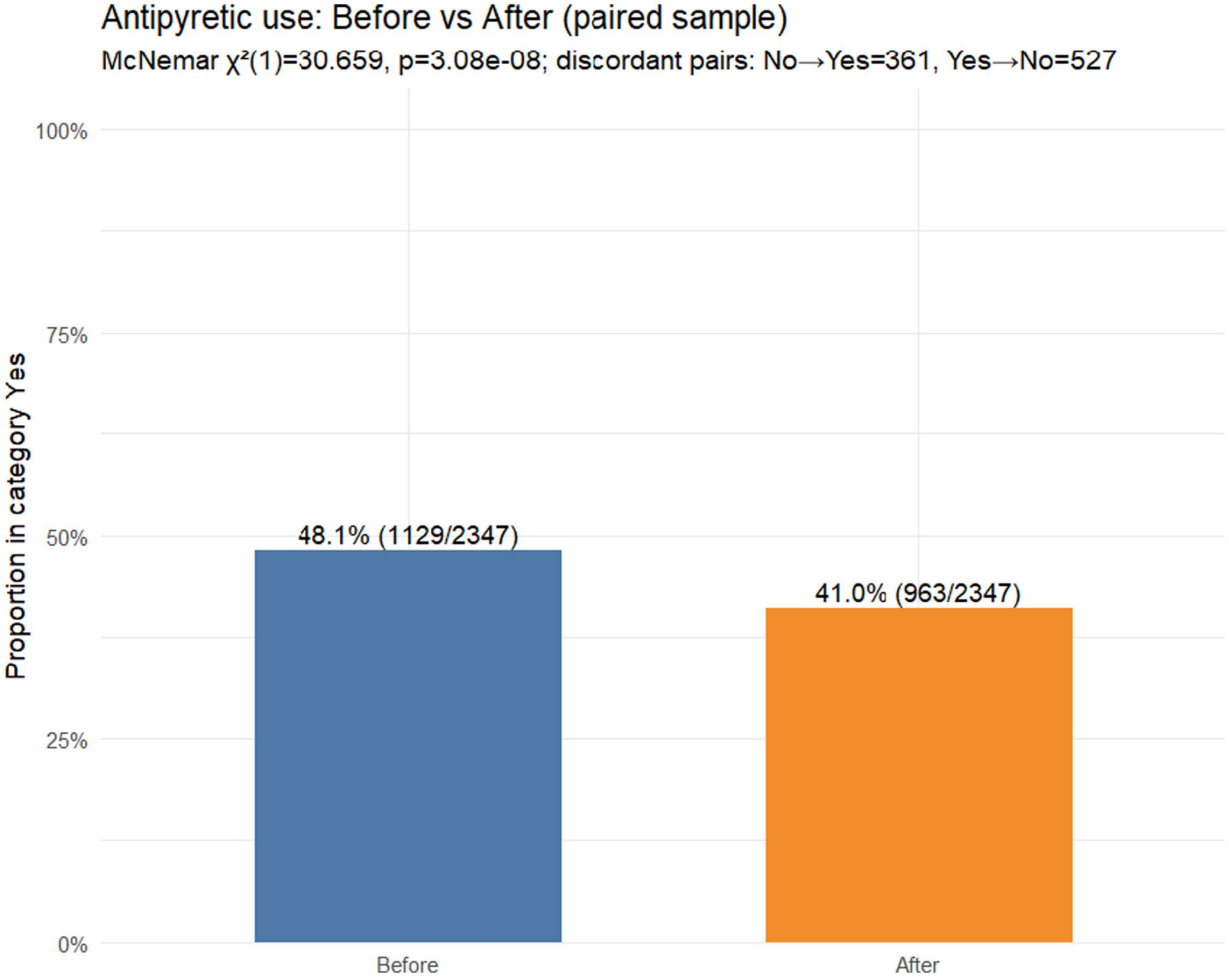
Antipyretic use before vs. after FeverFriend use (paired sample, *N* = 2,347).

In the paired sample (*N* = 2,347). Antipyretic use was reported at baseline (12-month recall) by 48.1% (1,129/2,347) compared with 41.0% (963/2,347) during the reviewed illness episode (Figure 3). Discordant pairs were *b* = 527 (yes → no) and *c* = 361 (no → yes), indicating a net shift towards less antipyretic use, corresponding to a paired risk difference of −7.1 percentage points (95% CI − 9.6 to −4.6). The paired change was statistically significant (McNemar’s *χ*^2^(1) = 30.659, *p* = 3.08 × 10^−8^).

*Hypothesis 3*: Changes in doctor visits due to fever

**Figure 4:**
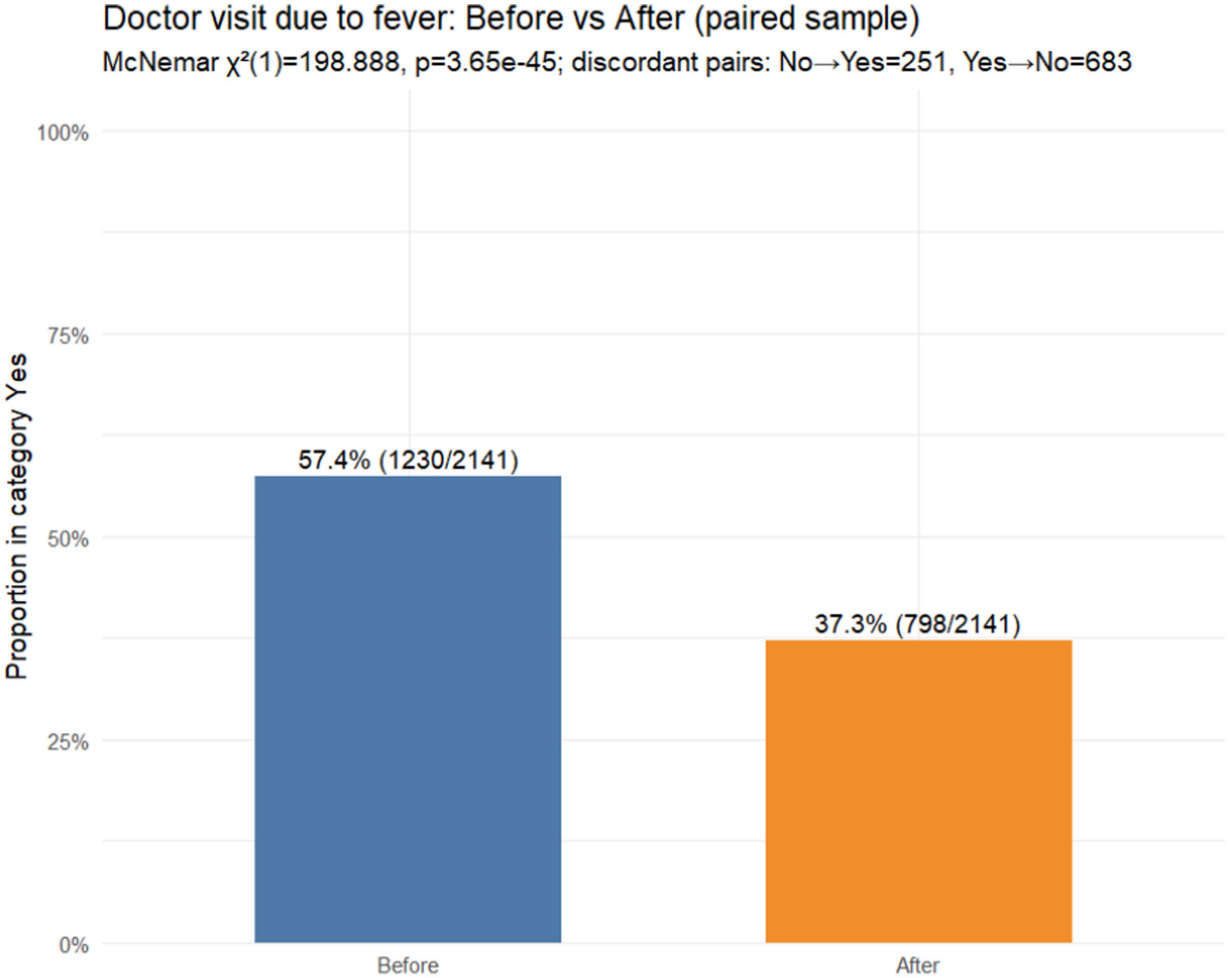
Doctor visits before vs. after FeverFriend use (paired sample, *N* = 2,141).

In the paired sample (*N* = 2,141), doctor consultation was reported at baseline (12-month recall) by 57.4% (1,230/2,141) compared with 37.3% (798/2,141) during the reviewed illness episode. Discordant pairs were *b* = 683 (yes → no) and *c* = 251 (no → yes), indicating a pronounced net shift towards fewer doctor consultations, corresponding to a paired risk difference of −20.2 percentage points (95% CI − 23.0 to −17.4). The paired change was highly significant (McNemar’s *χ*^2^(1) = 198.89, *p* < 3.65 × 10^−45^) (Figure 4).

The *p*-value, which is less than the usual significance level of 0.05, suggests strong evidence to reject the null hypothesis. Therefore, we conclude that there is a statistically significant association between baseline recall and episode-level reporting within the paired sample.

The results of the Bonferroni correction indicated that the use of antibiotics, antipyretics and medical consultation remained statistically significant after accounting for multiple testing.

Overall, our analysis provides statistically robust evidence of differences between baseline recall and episode-level reporting on the use of both antibiotics and antipyretics and doctor visits due to fever, even after considering multiple testing adjustments.

Regarding safety, no clinical complications were reported by caregivers in the reviewed episodes despite lower episode-level reporting of healthcare contacts. However, because complications were not systematically ascertained and follow-up was incomplete, these registry data do not allow evaluation of diagnostic accuracy (e.g., sensitivity/specificity) or safety outcomes beyond self-report.

## 4 Discussion and conclusion

The overuse of antibiotics and antipyretics in general pediatric practice and the associated risk of antimicrobial resistance (AMR) are important considerations in healthcare (29, 30). Sociological research attributes parental attitudes to feverish pediatric illnesses to a number of factors such as unjustified fear, help-seeking behavior, complex behavioral patterns of symptom avoidance and comfort-seeking. In the light of recent research, target points of improvement in attitudes are linked to healthcare literacy among health professionals and lay people. The role of the young parents’ generation using media is crucial. In response, our program—including the publicly available FeverFriend mobile app containing a differential diagnostic algorithm as a decision aid and an integrated knowledge base—aims to promote evidence-based practice, resulting in reduced drug consumption as well as alleviating the burden on the healthcare system by unnecessary consultations.

With self-reporting EMA, real time and real-life circumstances were captured.

In summary, we can conclude that our sample, although not random, aligns well with relevant external sources, indicating good coverage of children in Hungary. The consistency observed in various characteristics, such as average number of children, age, gender distribution, weight, and height, strengthens the reliability of our sample and permits the assumption that the following measures on healthcare consumption could be generalized.

### 4.1 Principal findings

This study provides real-world registry data showing statistically significant differences between 12-month baseline recall and subsequent episode-level reporting among app users. These findings are consistent with an association between app use and lower episode-level reporting of antibiotics, antipyretics, and healthcare visits; controlled designs with comparable pre/post measurement windows are needed to assess effectiveness. These findings suggest that an integrative-supportive, digitally guided approach may positively influence caregiver decision-making while maintaining safety.

The analysis of healthcare consumption among app users revealed significant changes in behavior. Regarding hypothesis 1, 2 and 3: in the reviewed episode, caregivers reported lower frequencies of antibiotic use, antipyretic use, and doctor visits due to illness compared with their 12-month baseline recall. These findings are consistent with an association between app use and lower episode-level reporting of antibiotic use, antipyretic use, and healthcare consultations. Outcomes related to caregiver confidence, satisfaction, or guideline adherence were not directly measured in the present analyses but can hypothesize change in healthcare behavior.

Age-related spontaneous regression of childhood infection rates were not likely to occur in a detection bias regarding decreased doctors contact and antipyretic use, as the mean duration between registration and illness recording was less than 3 months and the average age at registration was 2.98 years.

### 4.2 Interpretation in the context of existing literature

The observed changes are consistent with previous research highlighting the role of caregiver anxiety, uncertainty, and health literacy in driving overmedicalization of childhood fever. Fever is frequently perceived as a harmful condition requiring immediate intervention, despite strong evidence supporting its physiological benefits. One plausible explanation is that risk stratification (RAG protocol), personalized guidance, and an accessible knowledge base may support caregiver decision-making and reduce decisional uncertainty; however, these mechanisms (e.g., health literacy, self-efficacy, guideline adherence) were not directly measured and should be considered hypotheses for future evaluation. This aligns with behavioral health models indicating that clear safety signals and actionable advice are key determinants of appropriate self-management.

The observed difference in behavioral change between the use of antipyretics compared to antibiotics (hypothesis 2) may be attributed to several factors. Firstly, antipyretics are available over the counter (OTC), allowing caregivers to make personal decisions regarding their use. In contrast, antibiotics are prescribed by medical doctors, limiting caregiver’s control over their administration. Additionally, after risk assessment, it has been found that most cases of childhood febrile illnesses can be managed with a delayed or no prescription strategy for antibiotics, resulting in satisfactory patient outcomes and uncomplicated clinical courses (31). Patients who contact healthcare providers more often, are more likely to receive antibiotic prescriptions (32). Therefore, interventions focusing on improving health literacy and fostering the dialog between patients and healthcare providers have the potential to decrease the overuse of antipyretics and antibiotics (33, 34, 35, 36, 37).

### 4.3 Strengths and limitations of the study

As the strength of the study, by collecting real-life and real-time data through the FeverFriend app with the EMA design, we were able to capture information on fever management and healthcare utilization as they naturally occurred in caregivers’ daily lives without notable respondent burden over a long period of time.

Secondly, our study benefited from a relatively large sample size, moreover, our study sample performed good coverage for the general Hungarian child population in the important dimensions enhancing the external validity of our findings.

Above data are also coherent with findings of the German FeverApp Registry (38).

It is important to acknowledge certain limitations of the study. One limitation is the comparison of a longer period of time (last 12 months before entry in the registry) with a specific shorter cross-sectional period (outcome of one illness) within a paired statistical test. This approach may introduce potential biases and confounding factors that could influence the observed results. Comparing a longer time period with a specific shorter one within a paired statistical test may not fully capture the dynamic nature of fever management practices. It is possible that the behavior and decision-making of caregivers during the last 12 months could vary over time and be influenced by different factors compared to those during a specific illness episode. Moreover, the baseline data referring to the last 12 months before entry in the registry may also be distorted by recall bias. Because ‘yes within 12 months’ is structurally more likely than ‘yes within one episode,’ paired differences cannot be interpreted as magnitude of change, nor as evidence of app efficacy. Future evaluations should use episode-matched pre/post comparisons (e.g., last illness episode before registration vs. first episode after registration) and/or controlled study designs.

Only a subset of episodes had a completed end-of-illness review, and paired analyses required both baseline and reviewed-episode data. We therefore conducted a descriptive child-level gap analysis (Table 1) comparing children with any complete paired data to children with baseline data only, using age, sex, and chronic disease status. This comparison showed no major observable differences in the available child-level variables.

Because some caregivers registered more than one child, observations may be correlated within caregiver accounts. The current paired analyses (McNemar tests) treat children as independent and therefore do not account for within-caregiver clustering; consequently, uncertainty estimates (and *p*-values) may be overly precise.

Additional sensitivity analyses addressing within-caregiver clustering and alternative episode-selection rules were beyond the scope of the present study. The findings should therefore be interpreted with reference to the analytic dataset and operational choice of the last reviewed episode used here.

App use intensity was not quantified in this analysis. Although the registry contains some process-related variables, such as numbers of measurements, numbers of recorded episodes, and timing of review completion, these were not considered adequate proxy measures of engagement because they are also influenced by illness course, recurrence, and review-completion behavior. As a result, we could not assess dose–response relationships or whether associations differed by engagement level. Future prospective evaluations should prespecify and validate engagement metrics that more directly capture interaction with the app and its knowledge resources.

### 4.4 Implications for practice and future research

Future analyses will incorporate methods that account for clustering (e.g., cluster-robust approaches or mixed-effects models) and/or sensitivity analyses restricted to one child per caregiver.

From a public health perspective, these findings are relevant to antimicrobial resistance (AMR) mitigation strategies. While most stewardship interventions focus on healthcare providers, this study demonstrates that caregiver-facing digital tools can function as upstream interventions, modifying health-seeking behavior before clinical encounters occur. By improving illness appraisal and supporting guideline-consistent home care, such tools may reduce unnecessary medical visits and downstream antibiotic prescribing pressure.

Furthermore, the app represents a scalable implementation strategy for integrative fever management, translating guideline recommendations into daily practice. Its integration into national emergency service platforms and clinical guidelines highlights its potential relevance for health-system–level adoption.

Future studies could consider incorporating a more balanced and consistent time frame for the comparison, such as comparing the baseline period with multiple fever episodes over a similar duration. This would provide a more comprehensive understanding of the long-term effects of the FeverFriend app on fever management behaviors.

Future research could focus on increasing user engagement and encouraging a higher rate of illness review within the app. This could be achieved through targeted interventions, reminders, or incentives to ensure a more comprehensive and representative dataset.

High antibiotic prescription rates by professional healthcare providers have to be investigated and captured by multimodal interventions to achieve a sustainable and sufficient improvement in guideline adherence and implementation in clinical practice.

In conclusion, this prospective app-based registry identified statistically significant differences between 12-month baseline recall and subsequent episode-level reporting of antibiotics, antipyretics, and doctor consultations among users of the FeverFriend app. These findings are consistent with an association between app engagement and patterns of fever management reporting. However, due to the observational design, self-selection, recall bias, and non-comparable measurement windows, the results should be interpreted as preliminary. Controlled or episode-matched studies should assess effectiveness and causal impact. The app seems to have the potential to improve adherence to latest guidelines and to enhance parental satisfaction with fever management. Future research should aim to investigate the long-term effects of the app and similar programs on fever management in the public healthcare system, as well as their potential to reduce overmedicalization and healthcare utilization.

## 5 Intellectual property

The name ‘FeverFriend’ and its associated decision-support algorithm are registered trademarks. The trademark is owned by the first author (HS). The FeverFriend application is publicly available free of charge. At the time of submission, there is no commercial licensing, revenue generation, or financial exploitation associated with the trademark. The intellectual property did not influence data collection, analysis, or interpretation.

## Data Availability

The original contributions presented in the study are included in the article/supplementary material, further inquiries can be directed to the corresponding authors.

## Appendix A

### Primary outcome measures

1. Fever events outcome, condition of the patient 1, body temperature grade Celsius/Fahrenheit
2. Fever events outcome, condition of the patient 2, duration of fever in days
3. Fever events outcome, condition of the patient 3, medication, vaccination type and dosage
4. Fever events outcome, condition of the patient 4, hydration: normal—somewhat decreased—severely decreased
5. Fever events outcome, condition of the patient 5, ventilation: rate per minute
6. Fever events outcome, condition of the patient 6, ventilation: wheezing and dyspnea (subjective scale 1–2–3-4-5; minimum 1; maximum 5; 1 is no symptom = better, 5 is the worst)
7. Fever events outcome, condition of the patient 7, skin condition: color and rash
8. Fever events outcome, condition of the patient 8, pulse rate beat per minute
9. Fever events outcome, condition of the patient 9, crying quality (no, normal, abnormal)
10. Fever events outcome, condition of the patient 10, eating last time in hours, vomiting (yes–no)
11. Fever events outcome, condition of the patient 11, urination painful or smelly (yes–no)
12. Fever events outcome, condition of the patient 12, awareness: normal—sleepy—no (subjective scale; minimum = best = normal; maximum = worst = no)
13. Fever events outcome, condition of the patient 13, exotic trip in the last 12 month (yes–no)
14. Fever events outcome, condition of the patient 14, seizure (yes–no)
15. Fever events outcome, condition of the patient 15, wry neck (yes–no)
16. Fever events outcome, condition of the patient 16, pain (yes–no) pain location local—general duration hours
17. Caregivers state 1, Caregivers own feeling about the progress of patients’ illness (subjective scale,1 best = optimal—2 = not sure—3 = worst = very worried)
18. Caregivers state 2, Caregivers personal opinion about patients’ state: (subjective scale: minimum = best = not severe, middle = somewhat severe, maximum = worst = very severe)
19. Care-givers state 3, Caregivers self-confidence in handling the situation (subjective scale: maximum = confident; worse = somewhat confident, even worse = not really, minimum = worst = not at all)
20. Action taken, evaluation 1, Use of medication, use of medical providers’ service
21. Action taken, evaluation 2, Use of medication no–yes (type and dosage)

### Other outcome measures

Baseline characteristics (profile of patient) 1, date of birth.

Baseline characteristics (profile of patient) 2, gender: male–female–other.

Baseline characteristics (profile) 3, height centimeter.

Baseline characteristics (profile) 4, weight kilogram.

Baseline characteristics (profile) 5, chronic disease yes–no, if yes: type.

Baseline characteristics (profile) 6, Former use of medication in the last 12 month (antipyretics, antibiotics) yes–no, number.

Baseline characteristics (profile) 7, Former use of healthcare providers service in the last 12 month no–yes, number of events and type of institution.

Baseline characteristics (profile) 8, Caregivers former state/self-confidence at fever events of the patient in the last 12 month: caregivers state of mind, level of anxiety, behavior (subjective scale 1-2-3-4)

## Appendix B

### Advice to the caregiver in the different patient conditions along the risk assessment (RAG protocol)

Red: The patient’s condition presents a serious, high risk. Consult a doctor immediately!

Amber: [patientName] should be carefully monitored and cared for. This optimally running fever should not be reduced. It is not the goal to achieve a fever-free body temperature. Please complete the status survey again not later than 12 h. If [patientName]’s condition worsens over the next 6–12 h contact your doctor. If [patientName] does not improve after 3 days from now, see your doctor. About the interventions, see the knowledge base.

Green: [patientName] is fine. No medical examination or intervention is needed. Home care and observation for the next 3 days is sufficient. The fever should not be reduced. It is not the goal to achieve a fever-free body temperature. Complete a health check if [patientName]’s condition worsens, but not later than 24 h.

## References

[1] Kerdar SH Himbert C Martin DD Jenetzky E. Cross-sectional study of parental knowledge, behaviour and anxiety in management of paediatric fever among German parents. BMJ Open. (2021) 11:e054742. doi: 10.1136/bmjopen-2021-054742 PMC8524294 34663671

[2] Hasday JD Thompson C Singh IS. Fever, immunity, and molecular adaptations. Compr Physiol. (2014) 4:109–48. doi: 10.1002/cphy.c130019 24692136

[3] Evans SS Repasky EA Fisher DT. Fever and the thermal regulation of immunity: the immune system feels the heat. Nat Rev Immunol. (2015) 15:335–49. doi: 10.1038/nri3843 25976513 PMC4786079

[4] Lin C Zhang Y Zhang K Zheng Y Lu L Chang H . Fever promotes T lymphocyte trafficking via a thermal sensory pathway involving heat shock protein 90 and α4 integrins. Immunity. (2019) 50:137–151.e6. doi: 10.1016/j.immuni.2018.11.013 30650373 PMC6432644

[5] Szőke H Bokkon I Martin D Jan V Ágnes K Zoltán K . The innate immune system and fever under redox control: a narrative review. Curr Med Chem. (2022) 29:4324–62. doi: 10.2174/0929867329666220203122239 35125078

[6] Schulman CI Namias N Doherty J Manning RJ Li P Elhaddad A . The effect of antipyretic therapy upon outcomes in critically ill patients: a randomized prospective study. Surg Infect. (2005) 6:369–75. doi: 10.1089/sur.2005.6.369 16433601

[7] Ahkee S Srinath L Ramirez J. Community-acquired pneumonia in the elderly: association of mortality with lack of fever and leukocytosis. South Med J. (1997) 90:296–8. doi: 10.1097/00007611-199703000-00006 9076300

[8] Krafft HS Raak CK Jenetzky E Zuzak TJ Längler A Martin D. Warming up for a better fever: a randomized pilot study in pediatric oncology. Pilot Feasibility Stud. (2022) 8:183. doi: 10.1186/s40814-022-01144-7 35974359 PMC9380316

[9] Newey C Skaar JR O’Hara M Miao B Post A Kelly T . Systematic literature review of the association of fever and elevated temperature with outcomes in critically ill adult patients. Ther Hypothermia Temp Manag. (2024) 14:10–23. doi: 10.1089/ther.2023.0004 37158862

[10] Bertille N Purssell E Corrard F Chiappini E Chalumeau M. Fever phobia 35 years later: did we fail? Acta Paediatr. (2016) 105:9–10. doi: 10.1111/apa.13221 26725574

[11] El-Radhi AS. Why is the evidence not affecting the practice of fever management? Arch Dis Child. (2008) 93:918–20. doi: 10.1136/adc.2008.139949 18562453

[12] El-Radhi ASM. Fever management: evidence vs current practice. World J Clin Pediatr. (2012) 1:29–33. doi: 10.5409/wjcp.v1.i4.29 25254165 PMC4145646

[13] Bakalli I Klironomi D Kola E Celaj E. The management of fever in children. Minerva Pediatr (Torino). (2022) 74:568–78. doi: 10.23736/S2724-5276.22.06680-0 35822579

[14] Wrotek S. Let fever do its job. Evol Med Public Health. (2020) 9:26–35. doi: 10.1093/emph/eoaa044 33738101 PMC7717216

[15] Möhler R Jenetzky E Schwarz S Gwiasda M Rathjens L Szoke H . Parental confidence in relation to antipyretic use, warning signs, symptoms and well-being in fever management—results from an app-based registry. Int J Environ Res Public Health. (2022) 19:14502. doi: 10.3390/ijerph192114502 36361379 PMC9654195

[16] van der Werf ET Duncan LJ Flotow PV Baars EW. Do NHS GP surgeries employing GPs additionally trained in integrative or complementary medicine have lower antibiotic prescribing rates? BMJ Open. (2018) 8:e020488. doi: 10.1136/bmjopen-2017-020488 PMC5875618 29555793

[17] Baars EW Belt-van Zoen E Breitkreuz T Martin D Matthes H von Schoen-Angerer T . The contribution of complementary and alternative medicine to reduce antibiotic use: a narrative review. Evid Based Complement Alternat Med. (2019) 2019:5365608. 30854009 10.1155/2019/5365608 PMC6378062

[18] Szőke HP Maródi M Vagedes J Székely B Magyarosi I Bedő A . The P.E.A.N.U.T. Method: update on an integrative system approach for chronic otitis media with effusion and adenoid hypertrophy in children. Antibiotics (Basel). (2021) 10:134. doi: 10.3390/antibiotics10020134 33573118 PMC7911258

[19] Baars EW Weiermayer P Szőke HP van der Werf ET. The introduction of the global traditional, complementary, and integrative healthcare research agenda on antimicrobial resistance. Antibiotics (Basel). (2025) 14:102. 39858387 10.3390/antibiotics14010102 PMC11762681

[20] Green C Krafft H Guyatt G Martin D. Symptomatic fever management in children: a systematic review of national and international guidelines. PLoS One. (2021) 16:e0245815. doi: 10.1371/journal.pone.0245815 34138848 PMC8211223

[21] Clinical Guideline of the Ministry of Interior on the Management of Febrile Conditions in Childhood Kovács T Katalin B Ferenc F Katalin H Zsuzsanna MÉ Zsófia E . A Belügyminisztérium egészségügyi szakmai irányelve a lázas állapot ellátásáról gyermekkorban. EGÉSZSÉGÜGYI KÖZLÖNY. (2025) 75:393–414. Available at: https://www.researchgate.net/publication/393472132_Guideline_of_the_ministry_of_interior_on_management_of_feverish_states_in_childhood_EGESZSEGUGYI_KOZLONY_2025#fullTextFileContent

[22] Fiebermanagement bei Kindern und Jugendlichen, Versorgung von akut auftretendem Fieber bei ansonsten gesunden Kindern und Jugendlichen im ambulanten Setting, S3-Leitlinie. Available online at: https://register.awmf.org/de/leitlinien/detail/027-074

[23] Fever in under 5s: assessment and initial management, NICE guideline (2019). Available online at: www.nice.org.uk/guidance/ng143 (Accessed November 26, 2021)

[24] Joosen C Toelen J Asscherickx W. Clinical decision support for parents through Mobile applications: a systematic assessment of pediatric fever management apps. Belg J Paediatr. (2024) 26:275–81.

[25] Shiffman S Stone AA Hufford MR. Ecological momentary assessment. Annu Rev Clin Psychol. (2008) 4:1–32. doi: 10.1146/annurev.clinpsy.3.022806.091415 18509902

[26] Trull TJ Ebner-Priemer UW. Ambulatory assessment. Annu Rev Clin Psychol. (2013) 9:151–76. doi: 10.1146/annurev-clinpsy-050212-185510 23157450 PMC4249763

[27] Fagerland MW Lydersen S Laake P. The McNemar test for binary matched-pairs data: mid-p and exact alternatives. BMC Med Res Methodol. (2013) 13:91. 23848987 10.1186/1471-2288-13-91 PMC3716987

[28] Schwarz S Martin DD Büssing A Kulikova O Krafft H Gwiasda M . Sociodemographic characteristics and interests of FeverApp users. Int J Environ Res Public Health. (2021) 18:3121. doi: 10.3390/ijerph18063121 33803541 PMC8002853

[29] de Jong J van den Berg PB de Vries TW de Jong-van den Berg LT. Antibiotic drug use of children in the Netherlands from 1999 till 2005. Eur J Clin Pharmacol. (2008) 64:913–9. doi: 10.1007/s00228-008-0479-5 18563402

[30] AMC | European Centre for Disease Prevention and Control. Available online at: https://qap.ecdc.europa.eu/public/extensions/AMC2_Dashboard/AMC2_Dashboard.html#geo-distribution-tab (Accessed June 18, 2023).

[31] Spinks A Glasziou PP Del Mar CB. Antibiotics for treatment of sore throat in children and adults. Cochrane Database Syst Rev. (2021) 12:CD000023. doi: 10.1002/14651858.CD000023.pub5 34881426 PMC8655103

[32] Cook R Davidson P White A. Clinicians prescribe antibiotics for childhood respiratory tract infection based on assessment, rather than parental expectation. BMJ. (2020):l6768. doi: 10.1136/bmj.l6768 31924664

[33] Spurling GK Mar CBD Dooley L Clark J Askew DA. Delayed antibiotic prescriptions for respiratory infections. Cochrane Database Syst Rev. (2017):9. doi: 10.1002/14651858.CD004417.pub5 PMC6372405 28881007

[34] Stenlund S Mâsse LC Stenlund D Sillanmäki L Appelt KC Koivumaa-Honkanen H . Do patients’ psychosocial characteristics impact antibiotic prescription rates? Antibiotics. (2023) 12:1022. doi: 10.3390/antibiotics12061022 37370341 PMC10295249

[35] Weniger Antibiotika. Available online at: https://www.weniger-antibiotika.de/ (Accessed November 18, 2025).

[36] Healthier Together. Available online at: https://www.healthiertogether.app/ (Accessed November 18, 2025).

[37] Alejandro AL Leo WWC Bruce M Gimutao K. Does antibiotic awareness campaigns exposure decrease intention to demand antibiotic treatment? Testing a structural model among parents in Western Australia. PLoS One. (2023) 18:e0285396. doi: 10.1371/journal.pone.0285396 37200317 PMC10194918

[38] Fever App. Available online at: https://www.feverapp.de/en/ (Accessed November 18, 2025).

